# Prognostic impact of examined lymph-node count for patients with esophageal cancer: development and validation prediction model

**DOI:** 10.1038/s41598-022-27150-6

**Published:** 2023-01-10

**Authors:** Shasha Yuan, Chen Wei, Mengyu Wang, Wenying Deng, Chi Zhang, Ning Li, Suxia Luo

**Affiliations:** 1 Department of Internal Medicine, The Affiliated Cancer Hospital of Zhengzhou University, Henan Cancer Hospital, No. 127 Dongming Road, Zhengzhou, 450008 Henan People’s Republic of China; 2 Department of Radiotherapy, The First Affiliated Hospital of Xinxiang Medical University, Xinxiang, Henan People’s Republic of China

**Keywords:** Cancer, Gastroenterology, Oncology

## Abstract

Esophageal cancer (EC) is a malignant tumor with high mortality. We aimed to find the optimal examined lymph node (ELN) count threshold and develop a model to predict survival of patients after radical esophagectomy. Two cohorts were analyzed: the training cohort which included 734 EC patients from the Chinese registry and the external testing cohort which included 3208 EC patients from the Surveillance, Epidemiology, and End Results (SEER) registry. Cox proportional hazards regression analysis was used to determine the prognostic value of ELNs. The cut-off point of the ELNs count was determined using R-statistical software. The prediction model was developed using random survival forest (RSF) algorithm. Higher ELNs count was significantly associated with better survival in both cohorts (training cohort: HR = 0.98, CI = 0.97–0.99, *P* < 0.01; testing cohort: HR = 0.98, CI = 0.98–0.99, *P* < 0.01) and the cut-off point was 18 (training cohort: *P* < 0.01; testing cohort: *P* < 0.01). We developed the RSF model with high prediction accuracy (AUC: training cohort: 87.5; testing cohort: 79.3) and low Brier Score (training cohort: 0.122; testing cohort: 0.152). The ELNs count beyond 18 is associated with better overall survival. The RSF model has preferable clinical capability in terms of individual prognosis assessment in patients after radical esophagectomy.

## Introduction

Esophageal cancer is an important global health problem with an ever-increasing prevalence^1,2^. It ranks seventh in annual incidence and sixth in global mortality amongst cancers, half of which occurs in China with a 5-year survival rate of > 20%^3–5^. Surgical resection is considered the standard of care in the management of patients with EC^6^. With decades of widespread, multicenter, clinical research, the classification of malignant tumors based on the tumor-node-metastasis (TNM) staging system, developed by the American Joint Committee on Cancer (AJCC), as well as sex and pathological type, are approved as the conventional independent prognostic indicators for EC^7,8^. Additionally, dissection of lymph nodes is performed during resection, and the pathologic status of the lymph nodes (LNs) significantly affects postoperative survival. However, opinions on the extent of lymphadenectomy necessary to maximize survival remains controversial^9,10^.

Owing to the lack of accurate early diagnostic approaches and effective prognostic indicators, the 5-year overall survival (OS) rate of EC is approximately 30%^11^. Currently, TNM staging is the main predictor of prognosis in patients with EC^12^. Nevertheless, the staging groups are inaccurate, leading to a range of survival outcomes for patients within the same stage group, which is not sufficient to provide accurate information regarding the prognosis of such patients^13^.

Thus, constructing a new prediction model is of utmost importance. Machine learning (ML) is being rapidly developed in the field of prediction because of promising and powerful algorithms involved^14^. The random survival forest (RSF) model is one of the most widely used methods of machine learning. The bootstrap method is used to randomly select samples and form multiple binary decision trees to form a random forest plot^15^.

In the present study, we investigated the relationship between ELNs and long-term survival and determined an optimal ELNs count threshold. By employing the Lasso-Cox regression model and the RSF algorithm, we identified a prognostic model that could accurately predict survival in patients with EC.

## Methods

### Patient population

We adopted a retrospective design by collecting data from two cohorts. One cohort comprised 734 patients with EC who underwent radical esophagectomy between January 2013 and November 2017 at the Affiliated Cancer Hospital of Zhengzhou University (Henan Cancer Hospital). The other cohort was identified from the Surveillance, Epidemiology, and End Results (SEER) cancer registry and included 3,208 patients with EC diagnosed between 2000 and 2015.

### Training cohort

We compiled a Chinese single-institutional registry of 734 consecutive instances of esophageal cancer that underwent surgical therapy with curative intent in the thoracic surgery department between January 2013 and November 2017. This study was approved by the Ethics Committee of the Henan Cancer Hospital (No. 2022-KY-0049-001).

The selection criteria were as follows: (1) pathologically confirmed diagnosis of EC and (2) surgical treatment with curative intent. We excluded patients who underwent palliative surgery. We equally ruled out surgical records with missing portions (including surgeries performed in a foreign hospital). Patients with a history of concomitant malignant diseases or other primary malignancies were also excluded. Moreover, patients were ruled out if they were defined as M1 preoperatively and lost to follow-up.

### Testing cohort

Patients were screened from the SEER program, a national database with information on all incident cancer cases in selected areas of the U.S., covering nearly 28% of the U.S. population^16^. We recruited 60,570 cases diagnosed between 2000 and 2015 from the testing cohort (covering 18 registries) using the SEER*Stat software (seer.cancer.gov/seer stat) Version 8.3.9.2. Data retrieved from the testing cohort included age at diagnosis, sex, histological type, grade, primary tumor site, AJCC T 7th edition, AJCC N 7th edition, survival months, vital status, and regional nodes examined. Analyses were restricted to cases defined by the International Classification of Diseases for Oncology (ICD-O-3)/World Health Organization 2008 site code C150 to C155. Patients were excluded if they had metastasis at diagnosis, other malignancies, or were not treated at the reporting facility. We equally excluded patients with missing, unknown, or invalid aspects of the following covariates: age, sex, histological type, grade, primary tumor site, T stage, N stage, survival months, vital status, and ELN count. Finally, 3,208 patients were enrolled in the study.

### Surgery and pathology

Surgical procedures included primary tumor resection and LN dissection. The McKeown or Sweet esophagectomy with radical lymphadenectomy was selected based on preoperative conditions and patient status. Surgery was performed by experienced doctors who can carry out complete lymph node dissection based on comprehensive preoperative examination using a unified model. All resected specimens were carefully examined by two senior pathologists following a uniform process. The number of LN was counted under a low-power field microscope. All processes were strictly and carefully executed to ensure the accuracy of lymph node count. The total number of lymph nodes was calculated as the total number of LNs resected in the cervical, thoracic, and abdominal regions. A pathological N stage was defined according to the eighth edition of the AJCC TNM classification system^17,18^.

### Follow-up

Patients were scheduled for follow-up every 3 months in the first 2 years after esophagectomy and every 6 months in the following years. The endpoint was death (disease-related or nonspecific) or the loss to follow-up. Disease-free survival (DFS) was defined as the time from surgery to first disease manifestation or death from any cause. Overall survival (OS) was evaluated as the time from surgery until death from any cause or last follow-up. The data of patients alive at the end of the study were censored for the purpose of analysis.

### Model development

The prediction model was developed in two stages: variable selection and model construction. The methods implemented at each stage and the prediction models are described below. We considered a variable selection method: Lasso regression analysis. A backward stepdown selection process based on the lowest Akaike information criterion (AIC) value was used in the Lasso-Cox regression model to make all variables in the model significant^19^. Next, the relationship between the selected variables and the outcome of interest was investigated using the Lasso-Cox regression model and RSF model. Random survival forest is an ensemble method, which uses the bootstrap sampling method to randomly select samples to form multiple binary survival trees, and then form a random survival forest plot^20^. The tree nodes are split according to the maximum survival difference between child nodes. For each bootstrap sample, approximately 37% of the samples in the training cohort were not extracted on average, and these samples were called out-of-bag (OOB) samples. The OOB error rate of the OOB sample was calculated and the lower the error rate, the better the model performance. For RSF model, the parameters ntree and node size were determined according to the lowest error rate using rsample package (the error rate = 31.7%, ntree = 500, node size = 10). Other parameters were set according to the default values.

### Assessment of model performance

The performance of the models was assessed based on the time-dependent area under the receiver operating characteristic curve (t-AUC). Model discrimination performance was determined using the Harrell concordance index (C-index). The C-index ranged from 0.5 (no better than chance) to 1.0 (perfect discrimination)^21^. The overall performance of the prediction model was quantified as the Brier score, reflecting the average squared deviation between the predicted probabilities for a set of events and their outcomes (0: perfect prediction and 1: completely false prediction). The prediction error curve can be used to graphically determine the prediction error of the Brier score over time^22^. The models were subjected to external testing with the SEER cohort.

### Statistical analyses

Continuous variables were presented as medians with interquartile ranges (IQR), while categorical variables were presented as percentages. Survival curves were plotted using the Kaplan–Meier method, and the log-rank test was used to assess differences in survival between the groups. The cut-off value of ELNs count in the training cohort was identified using R-statistical software and the survival package and was validated by analyzing the testing cohort. Probability (*p*) values < 0.05 and the statistical tests were based on a two-sided significance level. Lasso regression analysis was performed using the glmnet package in R-statistical software. RSF and stepwise selection models were implemented using the Random Forest SRC and MASS packages, respectively. All statistical analyses were performed using R version 4.2.0 (https://www.r-project.org/).

### Ethical approval

The study was conducted in accordance with the principles of the Declaration of Helsinki, the study protocol was approved by the ethics committee of affiliated cancer hospital of zhengzhou university (NO. 2022-KY-0049-001) and individual consents for this retrospective analysis were waived.

## Results

### Patient characteristics and distribution of ELNs number

We enrolled 743 and 3,208 patients with EC in the training and testing cohorts, respectively. The demographic characteristics and pathological findings for each cohort are presented in Table 1. The median follow-up time in the training cohort was 55.7 months (range: 0.9–103.23 months), and the 3- and 5-year survival rates were 68.5% and 45.5%, respectively. The 3- and 5-year recurrence rates were 58.7% and 39.8%, respectively. For the testing cohort, the median follow-up time was 39.00 months (range: 1.00–107.00 months), and the 3- and 5-year survival rates were 54.7% and 27.9%, respectively. The median ELN count was 27 (range, 4–117; mean, 28.52; SD, 10.917) in the training cohort and 14 in the testing cohort (range, 1–87; mean, 16.15; SD, 10.197). Supplementary Fig. 1 shows the frequency distribution of ELNs in both cohorts.

**Table 1:** Patient Characteristics.

Variables	Level	Single-database	Seer-database
		743	3208
Sex (%)	Male	539 (72.5)	2669(83.2)
	Female	204 (27.5)	539 (16.8)
Age (years, %)	< 50	58 (7.8)	226(7.0)
	50–60	209 (28.1)	756 (23.6)
	60–70	358 (48.2)	1369 (42.7)
	> 70	118 (15.9)	857 (26.7)
Tumor site (%)	Upper third	129 (17.4)	72 (2.2)
	Middle third	472 (63.5)	480 (15.0)
	Lower third	142 (19.1)	2656 (82.8)
Histological type (%)	SQC	690 (92.9)	620 (19.3)
	AC	53 (7.1)	2420 (75.4)
	Other	0 (0.0)	168 (5.2)
Grade (%)	Low differentiation	291 (39.2)	1510 (47.1)
	Middle differentiation	309 (41.6)	1439 (44.9)
	High differentiation	48 (6.5)	259 (8.1)
	Other	95 (12.8)	0 (0.0)
Pathologic T category (%)	T1	158 (21.3)	814 (25.4)
	T2	158 (21.3)	512 (16.0)
	T3	395 (53.2)	1764 (55.0)
	T4	32 (4.3)	118 (3.7)
Pathologic N category (%)	N0	438 (59.0)	1420 (44.3)
	N1	171 (23.0)	1224 (38.2)
	N2	101 (13.6)	432 (13.5)
	N3	33 (4.4)	132 (4.1)
ELNs count (median [IQR])		27.00 (21.00, 35.00)	14.00 (9.00, 21.00)

*SQC* Squamous-cell carcinoma, *AC* Adenocarcinoma, *AJCC* American Joint Committee on Cancer, *ELNs count* Examined lymph nodes count, *IQR* Interquartile range.

### Independent prognostic factors in the training cohort

After univariate analysis via Cox regression analysis, data on the variables of sex, tumor site, histological type, grade, pathologic T category, pathologic N category, and the ELNs count were entered into multivariable logistic regression analyses. However, histological type and grade were not found to be significant. Multivariate analyses demonstrated that hazard ratios were significantly higher for the factors of male sex, tumor site, advanced depth of invasion, increased number of metastasized lymph nodes, and decreased number of examined lymph nodes (Table 2).

**Table 2:** Multivariate Cox regression analysis of lymphadenectomy number on OS in the two cohorts.

Variables	Training cohort			Testing cohort		
	HR	CI	*P*	HR	CI	*P*
**Sex**	Ns			Ns		
Female	Ns			Ns		
Male	1.66	1.25–2.19	0.004	1.24	1.09–1.41	0.001
**Age (years)**						
< 50	Ns			Ref		
50–60	Ns			1.23	1.01–1.51	0.044
60–70	Ns			1.23	1.01–1.49	0.037
> 70	Ns			1.77	1.45–2.16	0.000
**Tumor site**						
Upper third	Ref			Ref		
Middle third	0.62	0.46–0.82	0.001	0.75	0.56–1.01	0.057
Lower third	0.43	0.29–0.63	0.000	0.65	0.49–0.87	0.003
**Histological type**						
AC	Ref			Ref		
SQC	1.15	0.75–1.75	0.5173	Ns		Ns
Other	Ns			Ns		Ns
**Grade**						
Low differentiation	Ref			Ref		
Middle differentiation	1.02	0.79–1.31	0.902	0.84	0.76–0.92	0.003
High differentiation	1.12	0.68–1.85	0.645	0.55	0.44–0.68	0.000
other	0.81	0.53–1.22	0.307			
**Pathologic T category**						
T1	Ref			Ref		
T2	1.28	0.86–1.91	0.228	1.46	1.24–1.72	0.000
T3	1.74	1.23–2.46	0.002	1.8	1.57–2.06	0.000
T4	1.63	0.92–2.89	0.095	1.86	1.45–2.38	0.000
**Pathologic N category**						
N0	Ref			Ref		
N1	2.69	2.05–3.53	0.000	1.26	1.13–1.4	0.000
N2	3.74	2.73–5.12	0.000	1.73	1.5–2	0.000
N3	5.91	3.97–9.23	0.000	3.35	2.71–4.13	0.000
ELNs count	0.98	0.97–0.99	< 0.001	0.98	0.98–0.99	0.000

*SQC* Squamous-cell carcinoma, *AC* Adenocarcinoma, *AJCC* American Joint Committee on Cancer, *ELNs count* Examined lymph nodes count.

### Impact of examined lymph-node number on survival and optimal count

**Figure 1:**
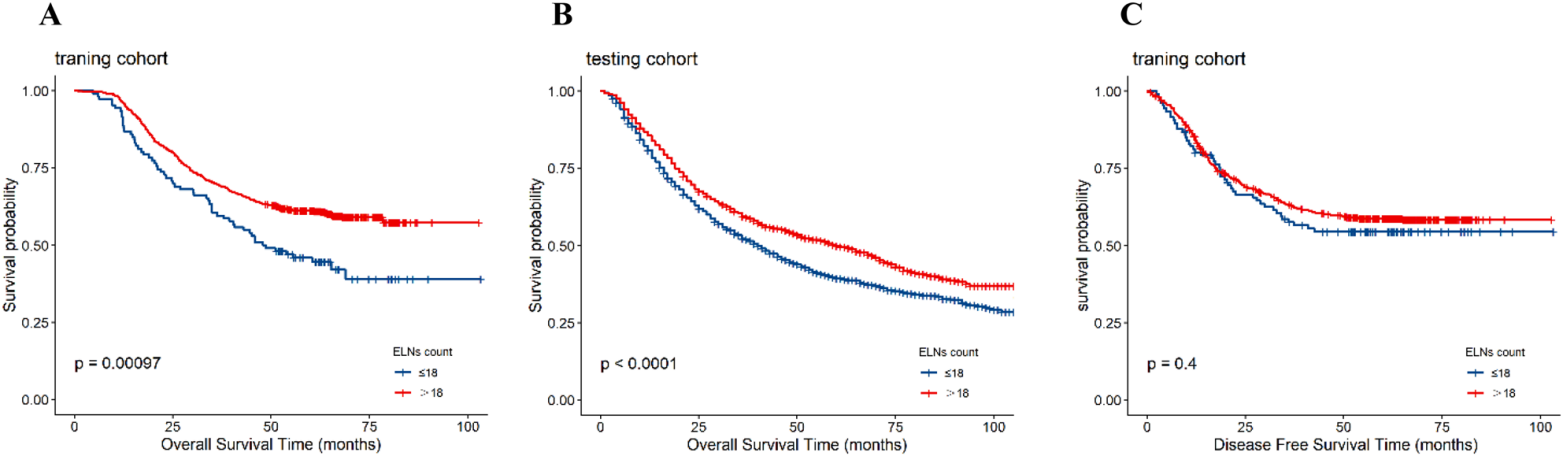
Overall Survival and Disease Free Survival of EC patients at the optimal ELNs count.

Table 2 reveals that an increasing ELN count was an independent factor favoring cancer survival (training cohort: HR = 0.98, CI = 0.97–0.99, *P* < 0.01; testing cohort: HR = 0.98, CI = 0.98–0.99, *P* < 0.01). Table 3 shows that the number of ELNs was an independent prognostic factor of DFS in the training cohort (HR = 0.99, CI = 0.97–1, *P* = 0.02). We determined that the optimal resected ELNs count was 18 in the training cohort using R-statistical software and the survival package. As shown in Fig. 1A, B, patients with resected ELN count > 18 had a better prognosis in both cohorts, whereas no significant difference was observed in the survival curves of DFS between the two groups in the training cohort (Fig. 1C).

**Table 3:** Cox regression analysis of lymphadenectomy number on DFS in single database.

Variables	Univariate			Multivariate		
	HR	CI	*P*	HR	CI	*P*
**Sex**				Ns		
Female	Ref			Ns		
Male	1.25	0.97–1.63	0.090	Ns		
**Age (years)**						
< 50	Ref			Ns		
50–60	0.86	0.56–1.32	0.481	Ns		
60–70	0.85	0.56–1.28	0. 432	Ns		
> 70	0.83	0.52–1.34	0.454	Ns		
**Tumor site**						
Upper third	Ref			Ref		
Middle third	0.92	0.68–1.24	0.584	Ns		
Lower third	0.88	0.61–1.29	0.520	Ns		
**Histological type**						
AC	Ref			Ref		
SQC	1.63	1.12–2.36	0.010	1.21	0.82–1.79	0.3391
Other	Ns			Ns		
**Grade**						
Low differentiation	Ref			Ref		
Middle differentiation	0.88	0.69–1.11	0.283	0.99	0.77–1.27	0.917
High differentiation	0.61	0.36–1.03	0.066	0.88	0.51–1.5	0.639
other	0.39	0.25–0.62	< 0.001	0.56	0.35–0.9	0.017
**Pathologic T category**						
T1	Ref			Ref		
T2	1.54	1.05–2.28	0.028	1.36	0.92–2.01	0.126
T3	2.05	1.48–2.85	< 0.001	1.49	1.06–2.11	0.022
T4	1.96	1.09–3.52	0.025	1.11	0.61–2.03	0.737
**Pathologic N category**						
N0	Ref			Ref		
N1	2.61	1.98–3.43	< 0.001	2.46	1.87–3.25	0.000
N2	3.89	2.9–5.24	< 0.001	3.44	2.52–4.72	0.000
N3	4.59	2.96–7.13	< 0.001	4.11	2.59–6.52	0.000
ELN counts	0.99	0.98–1	0.066	0.99	0.97–1	0.019

*SQC* Squamous-cell carcinoma, *AC* Adenocarcinoma, *AJCC* American Joint Committee on Cancer, *ELN counts* Examined lymph node counts, *PLN counts* Positive lymph node counts.

### Subgroup analyses

In T1, T2 ,and T3 + T4 cases, we noted that patients having beyond 18 inspected nodes had greater overall survival rates (Supplementary Fig. 2 A–F) in both cohorts. Since few patients had T4 tumors, we merged T3 and T4 into one T3 + T4 group. The same finding was only observed in N0-1 stages (Supplementary Fig. 3A, C vs. B, D) in both cohorts. In the histologic type subgroup analysis, the ELN count (ELNs > 18 vs. ELNs ≤ 18) was an independent prognostic factor of OS in squamous cell carcinoma stages, but not in adenocarcinoma in the training cohort (Supplementary Fig. 4A, B), whereas in the validation cohort, the result was observed in both squamous cell carcinoma and adenocarcinoma (Supplementary Fig. 4C, D). Owing to the limited data collected from the database, we only performed subgroup analysis on preoperative and postoperative treatment on the data of the training cohort. The survival benefit of lymph node dissection greater than 18 was only found in patients who did not receive preoperative or postoperative adjuvant therapy (Supplementary Fig. 5B, D vs. A, C).

### Model development

The design of the model is illustrated in Supplementary Fig. 6. The process of screening variables using Lasso regression analysis (with no zero coefficients) is shown in Supplementary Fig. 7A, B. Five statistically significant variables were retained in the Lasso-Cox and RSF models: sex, tumor site, pathological T category, pathological N category, and ELN count. In addition, we developed models containing the TNM stage to compare the RSF model with the AJCC stage.

### Model performance

**Figure 2:**
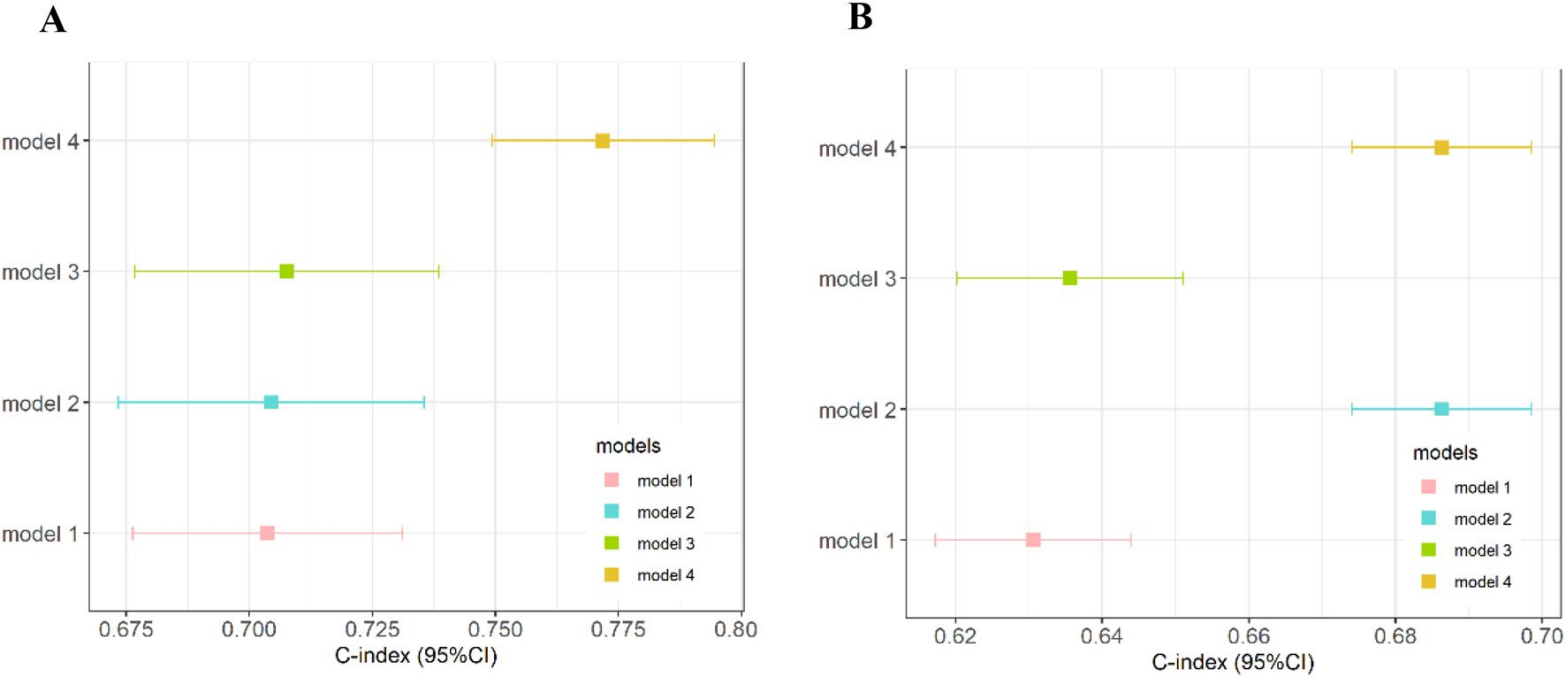
C-index of the prediction models. (**A**): Training cohort; (**B**): Testing cohort; model 1, Cox-TNM; model 2, Lasso-Cox; model 3, RSF-TNM; model 4, RSF.

**Figure 3:**
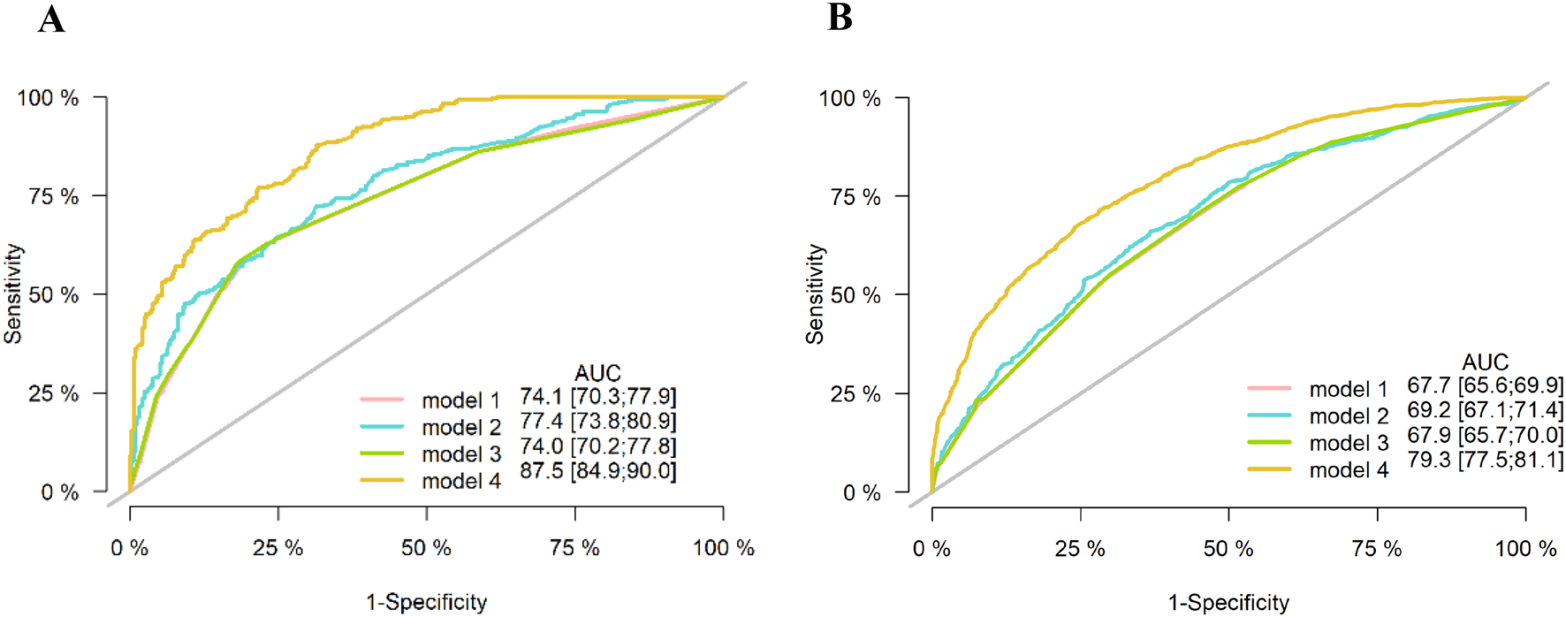
ROC curves of the prediction models. (**A**): Training cohort; (**B**): Testing cohort; model 1, Cox-TNM; model 2, Lasso-Cox; model 3, RSF-TNM; model 4, RSF.

**Figure 4:**
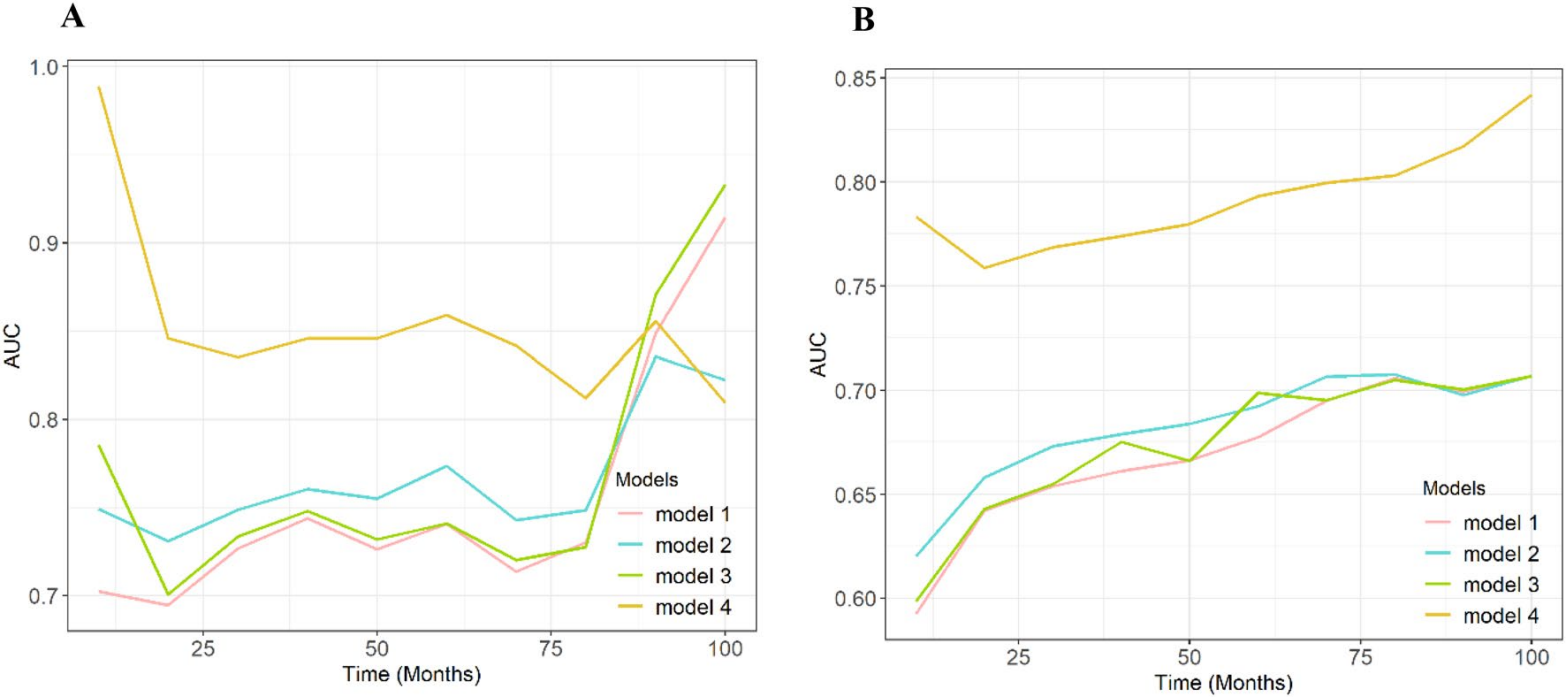
AUC curves of the prediction models. (**A**): Training cohort; (**B**): Testing cohort; model 1, Cox-TNM, model 2, Lasso-Cox; model 3, RSF-TNM; model 4, RSF.

**Figure 5:**
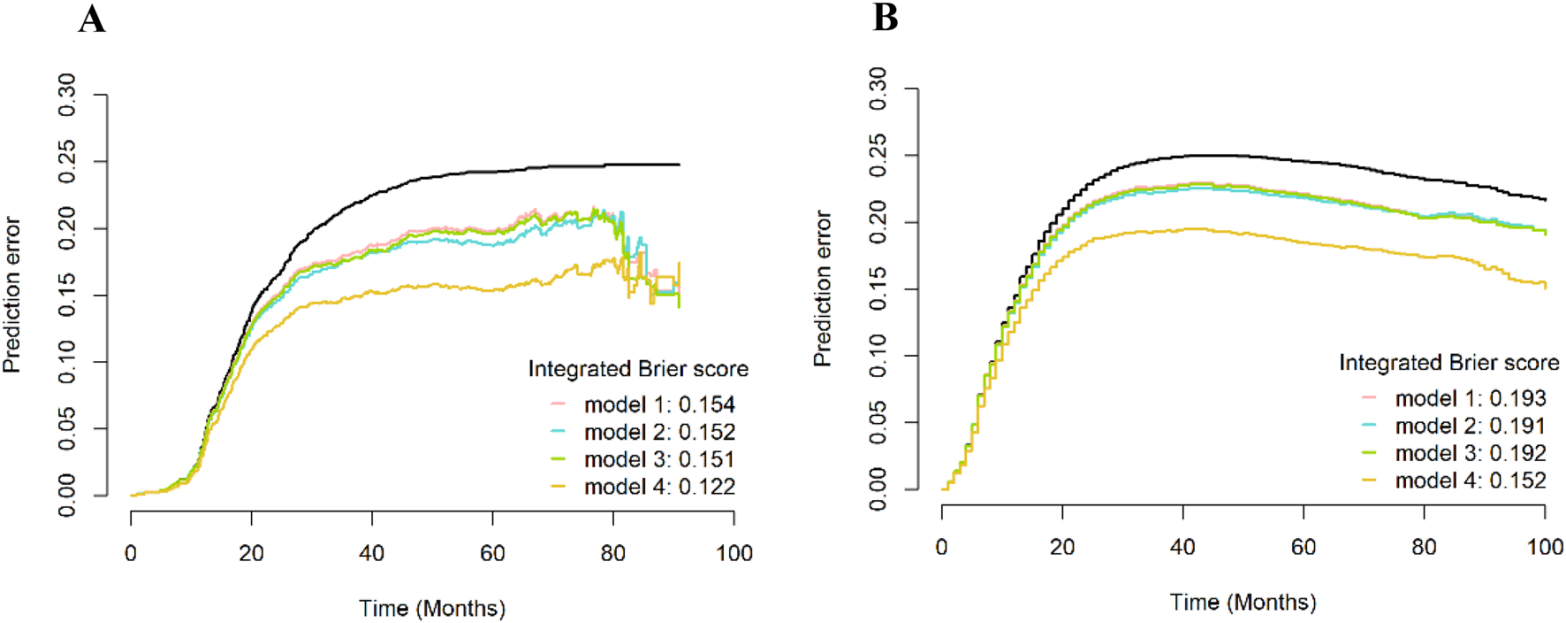
Prediction error curves of the prediction models. (**A**): Training cohort; (**B**): Testing cohort; model 1, Cox-TNM; model 2, Lasso-Cox; model 3, RSF-TNM; model 4, RSF.

Figure 2A, B illustrates the discrimination of the model assessed using the C-index. The C-index of the RSF model was highest among the four models in both the training and testing cohorts. Figure 3A, B shows the ROC curves for the different models. Moreover, the RSF had the highest AUC (Cox-TNM vs. Lasso-Cox vs. RSF-TNM vs. RSF: training cohort: 74.1 vs. 77.4 vs. 74.0 vs. 87.5.; testing cohort: 67.7 vs. 69.2 vs. 67.9 vs. 79.3). In addition, we plotted time against AUC curves for each model (Fig. 4A, B). We found that the AUC changed over time. Figure 5A, B shows the prediction error curves of the models. In both cohorts, the RSF model had lowest prediction error curve, reflected by a smallest Brier Score (Cox-TNM vs. Lasso-Cox vs. RSF-TNM vs. RSF: training cohort: 0.154 vs. 0.152 vs. 0.151 vs. 0.122.; testing cohort: 0.193 vs. 0.191 vs. 0.192 vs. 0.152).

## Discussion

In this study, we demonstrated that the number of retrieved lymph nodes removed was significantly associated with a favorable prognosis in patients with EC from both cohorts. This conclusion corroborated with the aforementioned findings^23^. Furthermore, we found the same correlation between ELNs and DFS. An optimal number of 18 resected lymph nodes demonstrated an improved OS but not DFS for patients who underwent esophagectomy. We tested the value of 18 using the SEER database and found that there were substantial differences between the cut-off values and the survival of patients with EC after esophagectomy. According to the theory: the long-term benefit is more important than the early endpoint^24^. We proposed 18 as the optimal number of lymph nodes in view of resection.

We further examined the relationship between ELNs and survival in different types of tumors. A higher number of ELNs had a positive effect on the OS in the T1, T2, and T3 + T4 stage tumors. Patients with a greater number of ELNs had improved OS in N0-1, but not in N2-3. We found that with deeper tumor invasion or more positive lymph nodes, higher ELN counts was not an independent factor favoring OS. In other words, the improvement in ELNs for survival is limited to its number. In terms of histologic type, patients with large number of ELNs were associated with better survival in both adenocarcinoma and squamous cell carcinoma of the testing cohort; however, the relationship was found only in squamous cell carcinoma of the training cohort and not in adenocarcinoma. In addition to this, we found that when people undergo preoperative or postoperative adjuvant therapy, the relationship was dispersed. It may be owing to the effect of adjuvant therapy on patients’ survival. Further studies are required to explore the complex relationship between adjuvant therapy and resected lymph nodes and survival. As previously mentioned, we know that the ELN count has been shown to be a superior indicator of survival of EC patients.

In order to better predict postoperative survival of patients with esophageal cancer, we attempted to identify more indicators. However, prognostic factors in patients with EC are known to be complicated. To date, a rough assessment is usually made based on the influencing factors confirmed by previous studies such as TNM stage and tumor grade, but not through individual analysis and judgment^25^. In view of this, we identified factors that affect the survival and prognosis of patients with EC and developed a prediction model.

Through Lasso regression analysis, we identified sex, tumor site, T and N category, and number of retrieved nodes as independent prognostic factors. These findings were consistent with those of previous studies on survival risk factors for esophageal cancer^7,8^. Then, a Lasso-Cox model was constructed for predicting survival. The hazard ratios were significantly higher for the factors of male sex, tumor site, advanced depth of invasion, increased number of metastasized lymph nodes, and decreased number of examined lymph nodes.

The Cox proportional hazards regression algorithm is commonly used to design models as we did; however, the conditions for its applicability are subject to several restrictions, such as the inaccuracy of models caused by the deviation of independent variable selection methods^26^. Compared with traditional survival analysis methods, the random survival forest model is not constrained by the proportional risk assumption, log-linear assumption, and other conditions. The machine learning-based risk prediction model yielded more favorable discrimination and significantly better accuracy than did the traditional model in this study. The RSF model outperformed the Lasso-Cox model with a higher C-index. Besides, in order to evaluate whether the RSF model could improve the prognostic prediction compared to the TNM stage, we developed models containing the TNM stage, and the results indicate that the RSF model showed better discrimination and accuracy. Moreover, we calculated the AUC for time-specific ROC curves at continuous time points, and the dynamic AUC line was plotted to depict temporal changes in accuracy. It also had the lowest Brier Score and prediction error curve. The results showed that the RSF model had a higher accuracy than did the other prediction models.

The variables of the RSF model were evaluated by variable importance (VIMP). The VIMP showed that the N stage was the largest important factor for prognosis (Supplementary Fig. 8). The N stage, the number of metastasis nodes identified, depends significantly on the number of nodes retrieved. As shown in Table 2, ELN counts were an important prognostic factor for patients with esophageal cancer, and more than 18 nodes reduced the risk scores significantly. The possible reasons for this finding are that retrieving more lymph nodes makes it more likely that the potentially metastasized lymph nodes will be resected. Moreover, the number of retrieved nodes may reflect the adequacy of surgical, pathological, and institutional care, all of which tend to affect treatment outcomes^27,28^.

Our study exhibited several strengths, including a large sample size, independent validation in an external cohort of patients, and the use of a machine-learning-based statistical tool for prediction model. In this study, we collected real data from our center as a training set and data from a foreign database as an external validation set. In addition, our data volume is very large. These factors added to the credibility of our finding of the RSF model showing considerable accuracy and efficacy compared with the COX model. Our results could be used in promising clinical applications prospectively, such as patient counseling, convenient prognosis assessment, and individualized follow-up strategy formulation, promoting the combination of prognostic tools and clinical management for operable EC patients.

However, we also encountered certain limitations. First, this single-center, retrospective cohort exhibited selection bias, undermining the generalizability of the best model recommended in this study. Second, several potential factors (tumor size, inflammatory biomarkers, and genetic data) were not included in the survival analysis since data collected was inadequate. Third, the algorithm and predictive process of the random survival forest model could not be expressed by a conventional formula as a nonparametric model, thereby affecting the generalizability and applicability of research conclusions to a certain extent. Fourth, two possible biases could have resulted in a miscount of LN number. These include underestimation as a result of the difficulty in separating each LN in the dissected tissues and overestimation because of fragmentation of nodal tissues during the removal of LNs, which might limit the application of a cut point. Further combined multicenter analyses should be considered, as well as prospective clinical verification of the precise value and a more acceptable cut-off number of lymph nodes.

In the present study, we found that a higher number of ELNs was associated with better prognosis, with an optimal ELN count of 18. In addition, we found the RSF model had the highest prediction accuracy among the four prediction models we developed. Thus, the RSF model is recommended for predicting the prognosis in patients with esophageal cancer after surgery.

## Supplementary Information

Supplementary Information 1.

Supplementary Information 2.

Supplementary Information 3.

Supplementary Information 4.

Supplementary Information 5.

Supplementary Information 6.

Supplementary Information 7.

Supplementary Information 8.

Supplementary Information 9.

## Data Availability

The database used in this study is publicly available and can be found in the SEER database (https://seer.cancer.gov/). And the datasets analyzed in this study are available from the corresponding author on reasonable request.

## References

[1] Ferlay J (2010). Estimates of worldwide burden of cancer in 2008: GLOBOCAN 2008. Int. J. Cancer.

[2] Nuytens F (2021). Five-year survival outcomes of hybrid minimally invasive esophagectomy in esophageal cancer: Results of the MIRO Randomized Clinical Trial. JAMA Surg..

[3] Jemal A (2011). Global cancer statistics. CA Cancer J. Clin..

[4] Smyth EC (2017). Oesophageal cancer. Nat. Rev. Dis. Primers.

[5] Bray F (2018). Global cancer statistics 2018: GLOBOCAN estimates of incidence and mortality worldwide for 36 cancers in 185 countries. CA Cancer J. Clin..

[6] Cloos-V Balen M (2022). Neoadjuvant chemoradiotherapy followed by resection for esophageal cancer: Clinical outcomes with the 'CROSS-regimen' in daily practice. Dis Esophagus.

[7] Semenkovich TR (2021). A clinical nomogram for predicting node-positive disease in esophageal cancer. Ann. Surg..

[8] Li X, Xu J, Zhu L, Yang S, Yu J, Lv W (2021). A novel nomogram with preferable capability in predicting the overall survival of patients after radical esophageal cancer resection based on accessible clinical indicators: A comparison with AJCC staging. Cancer Med..

[9] Herrera LJ (2010). Extent of lymphadenectomy in esophageal cancer: How many lymph nodes is enough?. Ann. Surg. Oncol..

[10] Mariette C, Piessen G (2012). Oesophageal cancer: How radical should surgery be?. Eur. J. Surg. Oncol..

[11] Pennathur A, Gibson MK, Jobe BA, Luketich JD (2013). Oesophageal carcinoma. Lancet.

[12] Fan Q, Liu B (2016). Identification of a RNA-Seq based 8-long non-coding RNA signature predicting survival in esophageal cancer. Med. Sci. Monit..

[13] Gupta V, Coburn N, Kidane B, Hess KR, Compton C, Ringash J (2018). Survival prediction tools for esophageal and gastroesophageal junction cancer: A systematic review. J. Thorac. Cardiovasc. Surg..

[14] Zhang X (2021). Aberrant functional connectivity and activity in Parkinson's disease and comorbidity with depression based on radiomic analysis. Brain Behav..

[15] Lin H, Zeng L, Yang J, Hu W, Zhu Y (2021). A machine learning-based model to predict survival after transarterial chemoembolization for BCLC stage B hepatocellular carcinoma. Front. Oncol..

[16] Doll KM, Rademaker A, Sosa JA (2018). Practical guide to surgical data sets: Surveillance, epidemiology, and end results (SEER) database. JAMA Surg..

[17] Rice TW (2016). Recommendations for pathologic staging (pTNM) of cancer of the esophagus and esophagogastric junction for the 8th edition AJCC/UICC staging manuals. Dis. Esophagus.

[18] Rice TW (2016). Recommendations for neoadjuvant pathologic staging (ypTNM) of cancer of the esophagus and esophagogastric junction for the 8th edition AJCC/UICC staging manuals. Dis. Esophagus.

[19] Xu C (2017). Socioeconomic factors and survival in patients with non-metastatic head and neck squamous cell carcinoma. Cancer Sci..

[20] Ishwaran H, Kogalur UB, Blackstone EH, Lauer MS (2008). Random survival forests. Ann. Appl. Stat..

[21] Matsui H, Fushimi K, Yasunaga H (2015). Variation in risk-standardized mortality of stroke among hospitals in Japan. PLoS ONE.

[22] Madjar K, Zucknick M, Ickstadt K, Rahnenführer J (2021). Combining heterogeneous subgroups with graph-structured variable selection priors for Cox regression. BMC Bioinform..

[23] Cao J (2016). Clinical nomogram for predicting survival of esophageal cancer patients after esophagectomy. Sci. Rep..

[24] Wilson MK (2015). Outcomes and endpoints in cancer trials: Bridging the divide. Lancet Oncol..

[25] Xie Z, Zhou H, Wang L, Wu Y (2022). The significance of the preoperative lactate dehydrogenase/albumin ratio in the prognosis of colon cancer: A retrospective study. PeerJ.

[26] Yang H (2021). Application of extreme learning machine in the survival analysis of chronic heart failure patients with high percentage of censored survival time. Front. Cardiovasc. Med..

[27] de Burlet KJ, van den Hout MF, Putter H, Smit VT, Hartgrink HH (2015). Total number of lymph nodes in oncologic resections, is there more to be found?. J. Gastrointest. Surg..

[28] Xia W (2019). Effect of lymph node examined count on accurate staging and survival of resected esophageal cancer. Thorac. Cancer.

