# Supplementary material for: Prognostic impact of examined lymph-node count for patients with esophageal cancer: development and validation prediction model
Source: Sci Rep. 2023 Jan 10;13:476. doi: 10.1038/s41598-022-27150-6 (PMC9831985; doi:10.1038/s41598-022-27150-6)
Supplement: Supplementary file 1 — Supplementary Information 1. [file 41598_2022_27150_MOESM1_ESM.docx]

**Supplementary** **Figure 1**. Distribution of the number of harvested lymph nodes in two cohorts. (ELNs count: examined lymph nodes count.)

and the SEER database. ELN, examined lymph node.


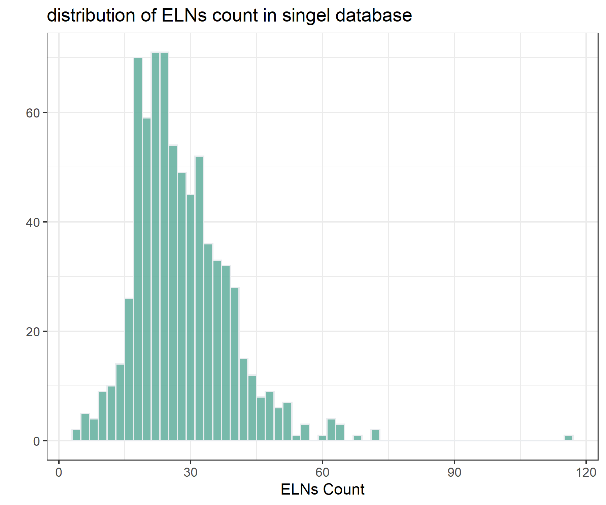

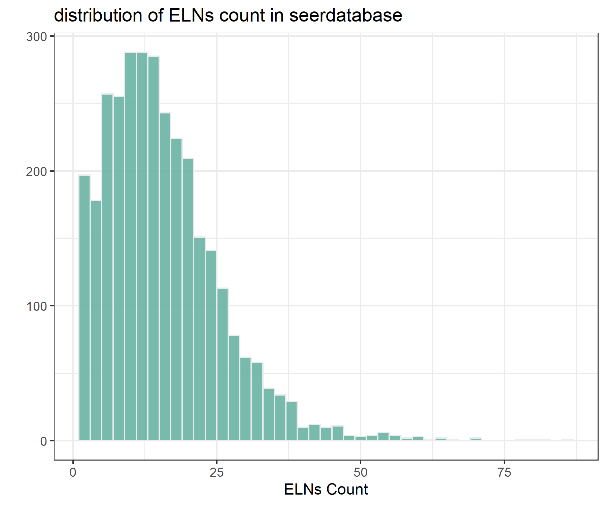


**B**

**A**
