# Supplementary material for: Prognostic impact of examined lymph-node count for patients with esophageal cancer: development and validation prediction model
Source: Sci Rep. 2023 Jan 10;13:476. doi: 10.1038/s41598-022-27150-6 (PMC9831985; doi:10.1038/s41598-022-27150-6)

**Supplementary Figure 5** Stratification of overall survival among patients with different preoperative and postoperative adjuvant therapy at the optimal ELNs count in training cohorts

**A**

**B**

**C**

**D**


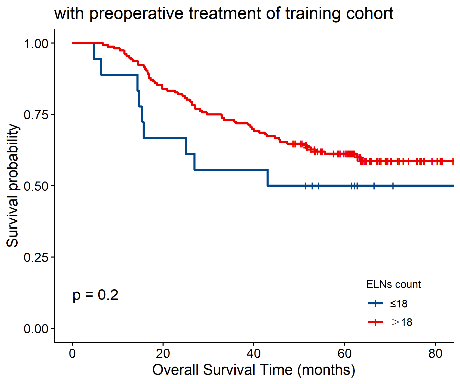

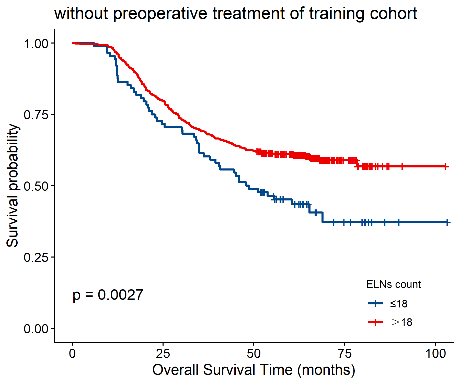

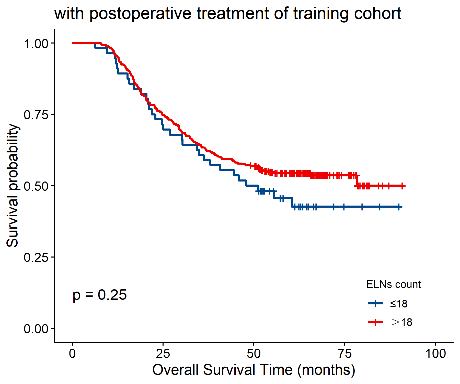

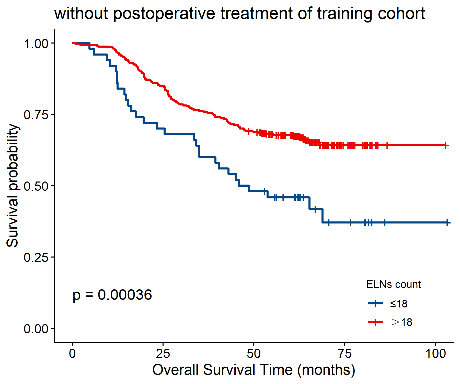

Supplement: Supplementary file 5 — Supplementary Information 5. [file 41598_2022_27150_MOESM5_ESM.docx]
