# Supplementary material for: Prognostic impact of examined lymph-node count for patients with esophageal cancer: development and validation prediction model
Source: Sci Rep. 2023 Jan 10;13:476. doi: 10.1038/s41598-022-27150-6 (PMC9831985; doi:10.1038/s41598-022-27150-6)
Supplement: Supplementary file 6 — Supplementary Information 6. [file 41598_2022_27150_MOESM6_ESM.docx]

Training cohort

(single database n=734)

Models including:

Cox-TNM model, Lasso-Cox model, RFS-TNM model

and RFS model

Model evaluation: ROC curve, t-AUC, C-index, prediction error

Feature selection

**Supplementary Figure 6** study design

Testing cohort

(SEER database n=3028)
