# Supplementary material for: Prognostic impact of examined lymph-node count for patients with esophageal cancer: development and validation prediction model
Source: Sci Rep. 2023 Jan 10;13:476. doi: 10.1038/s41598-022-27150-6 (PMC9831985; doi:10.1038/s41598-022-27150-6)

**Supplementary Figure 7** variables selection with the LASSO regression method. A: Tuning variable (lambda); B: A coefficient profile plot.

**A**

**B**


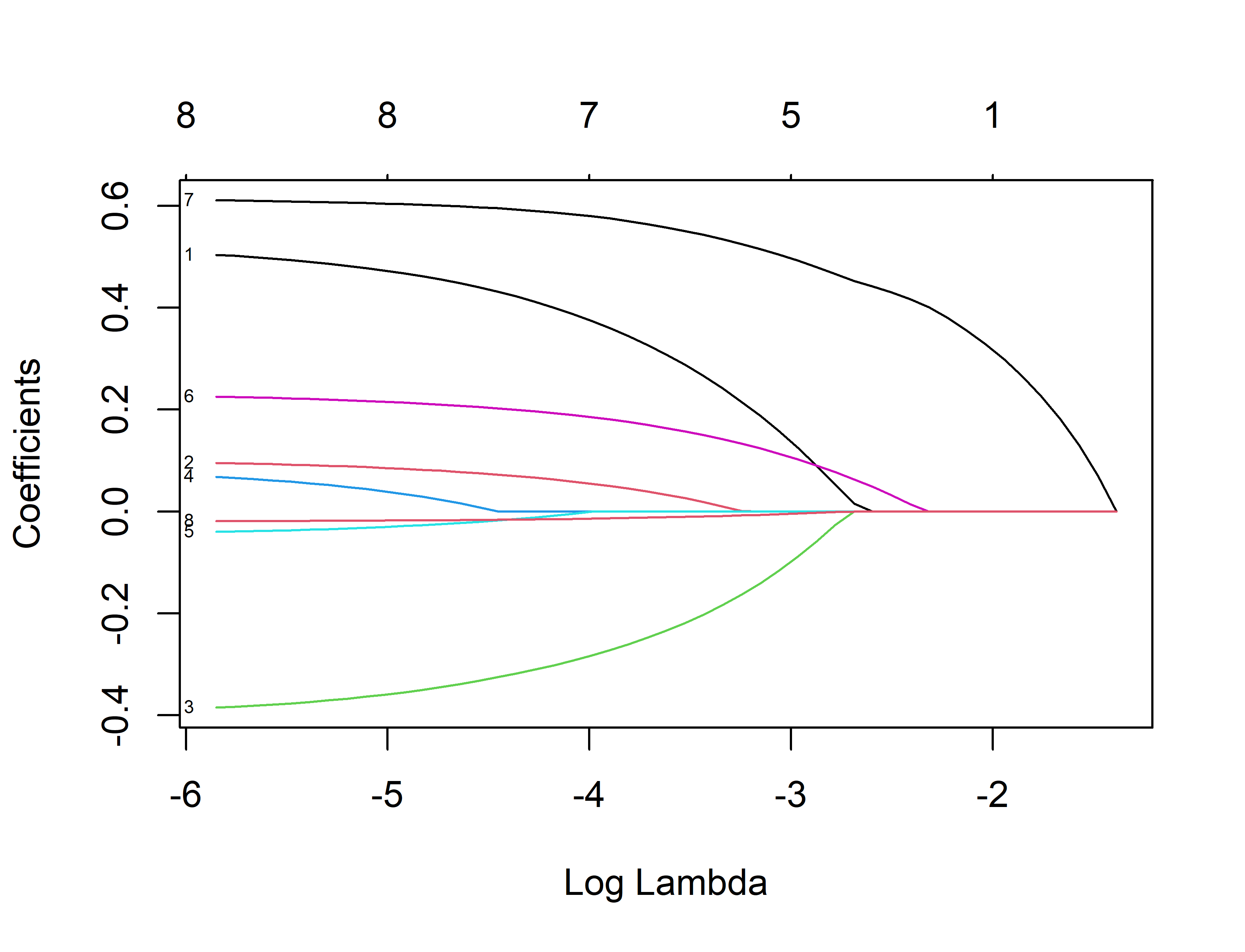

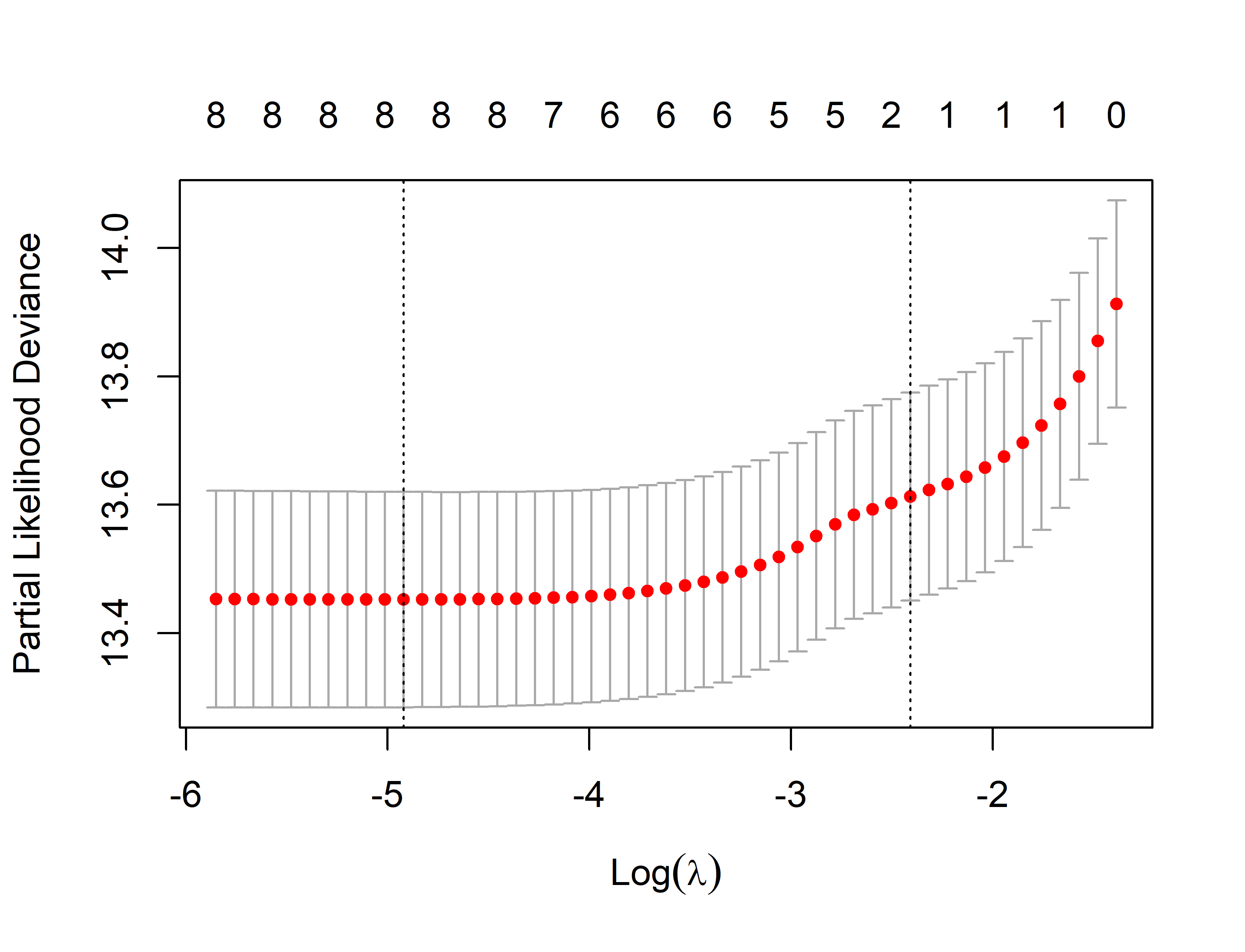

Supplement: Supplementary file 7 — Supplementary Information 7. [file 41598_2022_27150_MOESM7_ESM.docx]
