# Supplementary figures and images for: Prognostic impact of examined lymph-node count for patients with esophageal cancer: development and validation prediction model
Source: Sci Rep. 2023 Jan 10;13:476. doi: 10.1038/s41598-022-27150-6 (PMC9831985; doi:10.1038/s41598-022-27150-6)

**Supplementary Figure 8** variable importance (VIMP)


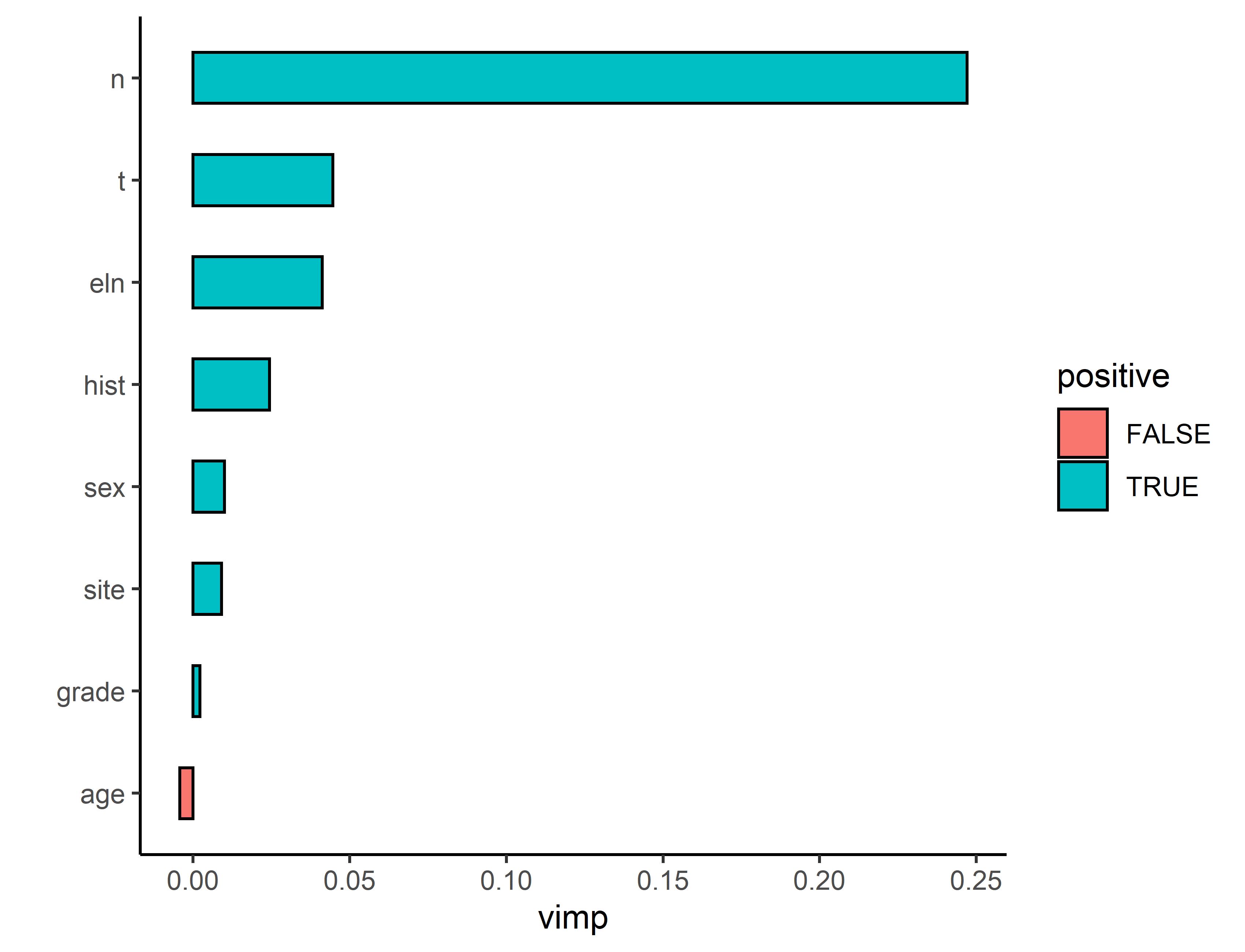

Supplement: Supplementary file 8 — Supplementary Information 8. [file 41598_2022_27150_MOESM8_ESM.docx]
