# Supplementary material for: Prognostic impact of examined lymph-node count for patients with esophageal cancer: development and validation prediction model
Source: Sci Rep. 2023 Jan 10;13:476. doi: 10.1038/s41598-022-27150-6 (PMC9831985; doi:10.1038/s41598-022-27150-6)
Supplement: Supplementary file 9 — Supplementary Information 9. [file 41598_2022_27150_MOESM9_ESM.docx]

**Supplementary Figure 1**. Distribution of the number of harvested lymph nodes in two cohorts. (ELNs count: examined lymph nodes count.)

**Supplementary Figure 2** Stratification of overall survival among patients with different T stages of esophageal cancer at the optimal ELNs count in two cohorts

**Supplementary Figure 3** Stratification of overall survival among patients with different N stages of esophageal cancer at the optimal ELNs count in two cohorts

**Supplementary Figure 4** Stratification of overall survival among patients with different histological types at the optimal ELNs count in two cohorts

**Supplementary Figure 5** Stratification of overall survival among patients with different preoperative and postoperative adjuvant therapy at the optimal ELNs count in training cohorts

**Supplementary Figure 6** study design

**Supplementary Figure 7** variables selection with the LASSO regression method. A: Tuning variable (lambda); B: A coefficient profile plot.

**Supplementary Figure 8** variable importance (VIMP)
